# Effects of natural extracts in cognitive function of healthy adults: a systematic review and network meta-analysis

**DOI:** 10.3389/fphar.2025.1573034

**Published:** 2025-03-27

**Authors:** Zhi-yuan Wang, Ya-lu Deng, Ting-yuan Zhou, Yi Liu, Yu Cao

**Affiliations:** Department of Emergency Medicine, Institute of Disaster Medicine and Institute of Emergency Medicine, West China Hospital, Sichuan University, Chengdu, China

**Keywords:** natural extract, diets, cognitive function, healthy adults, network meta-analysis

## Abstract

**Background:**

For years, diets and natural extracts have been explored for boosting cognition, but limited evidence challenges their recommendation for widespread use.

**Objective:**

Our study aimed to perform a network meta-analysis to evaluate effects of natural extracts on cognitive function in healthy adults. Methods: Researchers reviewed randomized controlled trials from Embase, PubMed, Cochrane Library, and Web of Science (up to 7 September 2024). Study quality was assessed with the Cochrane Risk of Bias Tool, and node-splitting analysis ensured consistency (p > 0.05). SUCRA values were calculated using parametric bootstrapping with 10,000 resamples. Primary outcomes included global cognition, attention, memory, executive function, and cognitive flexibility, with efficacy ranked by SUCRA probabilities.

**Results:**

From 27 studies with 2,334 samples and 19 natural extract treatments, RPTW showed the greatest improvement in overall cognition (SUCRA: 95.9%). No extracts significantly outperformed placebo for attention. CG (Cistanche + Ginkgo biloba) was most effective for memory (SUCRA: 89.3%), executive function (SUCRA: 96.9%), and cognitive flexibility (SUCRA: 98.0%).

**Conclusion:**

RPTW extracts improve overall cognition in healthy adults, while CG enhances memory, executive function, and cognitive flexibility.

**Systematic Review Registration:**

https://inplasy.com/inplasy-2024-11-0007/, identifier INPLASY (INPLASY2024110007).

## 1 Introduction

Cognitive function refers to the mental processes involved in reasoning, knowledge acquisition, and information processing, encompassing areas such as executive function, attention, cognitive flexibility, and memory(Harada et al., 2013). As life expectancy rises, maintaining cognitive function is crucial for healthy adults to make informed lifestyle choices and remain independent(Turrini et al., 2023). Cognitive decline, which often progresses to dementia(Bäckman et al., 2005; Karr et al., 2018), starts in early adulthood and accelerates during midlife(Harada et al., 2013; Schwarz et al., 2024; Massengale et al., 2024), significantly impacting the quality of life and wellbeing of adults at all ages(Schafer and Shippee, 2010).According to the 2019 Global Burden of Disease report, it is projected that by 2050, the number of dementia cases worldwide will increase to 152.8 million, which is 2.66 times higher than in 2019 (GBD, 2019 Dementia Forecasting Collaborators, 2022). However, no medications or treatments are available for cognitive decline currently. There is an urgent need to develop strategies to delay or even prevent the onset of cognitive impairment.

In recent years, there has been a growing interest in natural extracts as dietary ingredients for healthy adults due to potential neuroprotective properties and relatively low side-effect profiles compared to pharmaceutical drugs, particularly those rich in phytochemical compounds(Roe and Venkataraman, 2021). Natural extracts are substances derived from plants, herbs, or other natural sources through processes like solvent extraction or distillation, used either in their crude form or further purified to isolate specific bioactive compounds (Seidel, 2012). In the United States, nearly 23% of adults (58 million) use dietary ingredients from natural extracts to improve brain health or cognitive flexibility, while about 8% (9 million) believe these ingredients can reverse or delay dementia. Most adults consider dietary ingredients effective for maintaining cognitive function, with 50% believing natural extract may help reverse dementia(Mehegan and Rainville, 2019).Examples include Ginkgo biloba(Kaschel, 2011a), rosmarinic acid from rosemary(Araki et al., 2020), flavonoids from blueberries(Cheng N. et al., 2024), and diosgenin from yam(Tohda et al., 2017), all of which have been shown to possess anti-inflammatory, antioxidant, and neuroprotective properties that may benefit cognitive function.

While traditional meta-analyses suggest that Ginkgo biloba and Bacopa monnieri may improve clinical assessments with mild adverse events in patients with mild cognitive impairment(Basheer et al., 2022; Yang et al., 2016), a 2018 report noted that the variety of extract-based dietary ingredients has grown by nearly 3,500 products annually over the past 20 years, indicating exponential growth(Roe and Venkataraman, 2021). It is essential to identify the most suitable treatment among the various natural extract options. There is a lack of evidence-based guidance on which natural extracts are most effective for enhancing cognitive function. Network meta-analysis (NMA) evaluates the effectiveness of various interventions through direct and indirect comparisons, providing effectiveness rankings. Utilizing network meta-analysis allows us to compare multiple treatments (such as anthocyanin, rosmarinic acid, polyphenol extract from grapes and blueberries, Bacopa monnieri, Ginkgo biloba, Eriobotrya japonica, spearmint, diosgenin, etc.) both indirectly and directly, offering a ranking of effectiveness based on available clinical data. This approach will provide a clearer understanding of the potential of natural extracts in managing cognitive decline and will guide future research and clinical practice in the prevention and treatment of neurodegenerative diseases.

## 2 Materials and methods

This review strictly adhered to the guidelines outlined in the Preferred Reporting Items for Systematic Reviews and Meta-Analyses (PRISMA) and was duly registered in INPLASY (INPLASY2024110007).

### 2.1 Search strategy

A comprehensive search included electronic databases: PubMed, Embase, Cochrane Library and Web of Science from inception to 7 September 2024. The search strategy was carefully designed based on the PICOS principle. (P) Population: Healthy adults (≥18 years)with or without subjective cognitive decline. (I) Interventions: Natural extract interventions above 4 weeks. (C) Comparison: Control group was treated with placebo. (O) Outcomes: Outcomes of interest included global cognitive state, memory, attention, executive function, cognitive flexibility. (S) Study design: Randomized controlled trial (RCT). Taking PubMed as an example, Table 1 provides a detailed overview of the search strategy. The search included a combination of MeSH terms and free words. We also searched the reference lists of included studies and relevant reviews to identify additional potential studies. If necessary, we will contact the author for further information.

**TABLE 1:** Search strategy on PubMed.

#1	“Cognitive Dysfunction”[MeSH]
#2	(((((((((((((((((((((((((Cognitive Dysfunctions[Title/Abstract]) OR (Dysfunction, Cognitive[Title/Abstract])) OR (Dysfunctions, Cognitive[Title/Abstract])) OR (Cognitive Disorder[Title/Abstract])) OR (Cognitive Disorders[Title/Abstract])) OR (Disorder, Cognitive[Title/Abstract])) OR (Disorders, Cognitive[Title/Abstract])) OR (Cognitive Impairments[Title/Abstract])) OR (Cognitive Impairment[Title/Abstract])) OR (Impairment, Cognitive[Title/Abstract])) OR (Impairments, Cognitive[Title/Abstract])) OR (Mild Cognitive Impairment[Title/Abstract])) OR (Cognitive Impairment, Mild[Title/Abstract])) OR (Cognitive Impairments, Mild[Title/Abstract])) OR (Impairment, Mild Cognitive[Title/Abstract])) OR (Impairments, Mild Cognitive[Title/Abstract])) OR (Mild Cognitive Impairments[Title/Abstract])) OR (Cognitive Decline[Title/Abstract])) OR (Cognitive Declines[Title/Abstract])) OR (Decline, Cognitive[Title/Abstract])) OR (Declines, Cognitive[Title/Abstract])) OR (Mental Deterioration[Title/Abstract])) OR (Deterioration, Mental[Title/Abstract])) OR (Deteriorations, Mental[Title/Abstract])) OR (Mental Deteriorations[Title/Abstract])
#3	#1 OR #2
#4	“Herbal Medicine”[MeSH]
#5	((((((((Medicine, Herbal[Title/Abstract]) OR (Herbalism[Title/Abstract])) OR (Hawaiian Herbal Medicine[Title/Abstract])) OR (Herbal Medicine, Hawaiian[Title/Abstract])) OR (Medicine, Hawaiian Herbal[Title/Abstract])) OR (Laau Lapaau[Title/Abstract])) OR (La au Lapa au[Title/Abstract])) OR (La’au Lapa’au[Title/Abstract])
#6	#4 OR #5
#7	“Plant Extracts”[MeSH]
#8	((((((Extracts, Plant[Title/Abstract]) OR (Plant Extract[Title/Abstract])) OR (Extract, Plant[Title/Abstract])) OR (Herbal Medicines[Title/Abstract])) OR (Medicines, Herbal[Title/Abstract])
#9	#7 OR #8
#10	#6 OR #9
#11	#3 AND #10

### 2.2 Inclusion and exclusion criteria

The inclusion criteria utilized in present meta-analysis are defined within PICOS framework as follows: (1) Studies involving cognitive function of healthy adults; (2) Research where intervention group receives treatment with different natural extracts; (3) Comparison of the intervention measures with inactive controls (such as placebos, standard care, no treatment, or habitual diet); (4) Study reports must include one or more of the following outcomes: global cognitive state, executive function, memory, attention, cognitive flexibility.

The exclusion criteria were as follows: (1) Inability to obtain full text; (2) Absence of a control group in the study; (3) Conference papers, case analyses, review articles or previous systematic reviews and meta-analyses, case reports, review articles, letters, animal experiments, and reviews; (4) Studies with incomplete or unreported data.

### 2.3 Study selection

The use of Endnote software facilitated the screening and management of extensive literature. Two authors, Z.W. and Y.D., independently screened titles and abstracts, excluding duplicates and non-relevant literature types such as reviews, conference abstracts, correspondence, case reports, protocols, animal studies, and non-RCTs. This rigorous filtering ensured only relevant studies proceeded. They then re-evaluated the remaining abstracts against the inclusion and exclusion criteria before conducting an in-depth review of the included articles. Any disagreements were resolved through consultation with the third author, Y.L., to reach a consensus. Importantly, this process was conducted without restrictions on the literature’s release date or language, ensuring a comprehensive review.

### 2.4 Data extraction

Two researchers, Y.L. and T.Z., carried out a comprehensive and independent extraction of relevant data utilizing a standardized and meticulously pre-designed form. Any disagreements between responsible authors for extracting data were solved by consensus with the third reviewer. Recorded data on research features included: (1) the first author; (2) publication date; (3) country; (4) average age of patients; (5) number of patients; (6) intervention duration and dosage; (7) outcomes used to assess cognitive function (global state, executive function, memory, attention, cognitive flexibility) for healthy adults.

### 2.5 Risk of bias of individual studies

Two raters independently assessed the methodological quality of included studies using the Cochrane Bias Risk Assessment Tool for RCTs. Seven domains were considered: (1) randomized sequence generation, (2) allocation concealment, (3) blinding of participants and personnel, (4) blinding of outcome assessment, (5) incomplete outcome data, (6) selective reporting, and (7) other bias. Trials were categorized into three levels of risk of bias: low risk, high risk, and unclear risk (no reporting or missing information).

### 2.6 Data analysis

Natural extracts were considered as the intervention measure. All variables were considered continuous and expressed using mean values and standard deviations (SD). Due to the non-uniform units of outcome variables in some studies, continuous variables in the research was reported using 95% CI and standardized mean differences (SMD). To account for potential differences among studies, random-effects model was employed for analysis(Jackson et al., 2011). Stata MP15.0 was utilized, following the PRISMA network meta-analysis(NMA) guidelines(Moher et al., 2015; Shim et al., 2017), a Bayesian framework with Markov Chain Monte Carlo simulation was used for NMA. We utilized Stata software to generate a network diagram for various natural extracts. Each node in the network diagram represents a different natural extract intervention and control. The lines between nodes represent direct comparisons between interventions. The node size and connecting lines thickness was positively correlated with study quantity. To determine the ranking of interventions, we employed a parametric bootstrapping procedure with 10,000 resamples to calculate the ranking probabilities for all rankings and outcomes. We calculated the average ranking for each intervention and SUCRA values. Furthermore, we utilized node-splitting analysis to assess the consistency between indirect and direct comparisons. This method allowed us to evaluate the transitivity and consistency assumptions by comparing direct evidence with indirect evidence, with a p-value >0.05 indicating consistency (Salanti et al., 2011). Network funnel plots were constructed and assessed for symmetry to investigate potential publication bias(Higgins et al., 2012). To further evaluate the impact of studies with a high risk of bias, we conducted a sensitivity analysis by excluding these studies and re-running the network meta-analysis.

## 3 Results

### 3.1 Study selection

**FIGURE 1:**
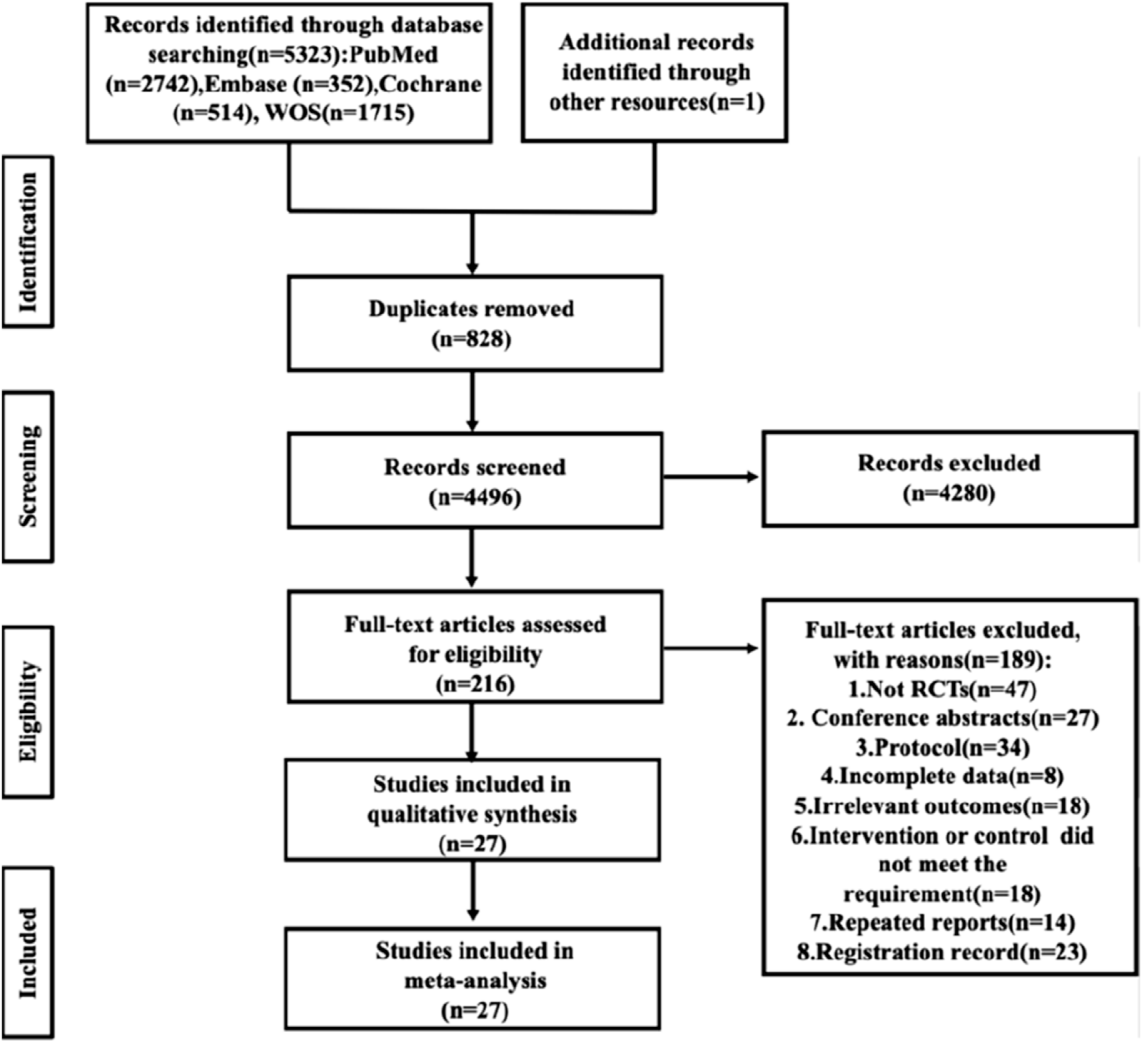
Preferred reporting items for systematic reviews and meta-analysis (PRISMA) flow diagram of systematic search and included studies.

During our database search, a total of 5,324 relevant articles were identified. The full texts of the remaining 218 articles were assessed for eligibility. Ultimately, 27 studies met our inclusion criteria and were included in the final analysis**(** Figure 1) (Araki et al., 2020; Cheng N. et al., 2024; Tohda et al., 2017; Ahles et al., 2020; Bell et al., 2022; Bensalem et al., 2019; Calabrese et al., 2008; Chai et al., 2019; Chen et al., 2024; Choi et al., 2016; Cieza et al., 2003; Crews et al., 2005; Fukuda et al., 2020a; Fukuda et al., 2020b; Hashimoto et al., 2022; Heuer et al., 2023; Kaschel, 2011b; Lee et al., 2020; Mix and Crews, 2002; Noguchi-Shinohara et al., 2023; Pipingas et al., 2008; Santos et al., 2003; Shin et al., 2009a; Wattanathorn et al., 2008; 2022; Whyte et al., 2018; Wong et al., 2012).

### 3.2 Characteristics and quality of included studies

A total of 27 RCTs were included in this meta-analysis, comprising 2,334 participants. Table 2 provides an overview of the characteristics of the included studies. The intervention group consisted of 1,231 participants, while the control group included 1,103 participants. These trials investigated 19 different interventions. The interventions included a variety of natural extracts such as Ginkgo biloba extract(GBE), tart cherry, cranberry, anthocyanin, rosmarinic acid, polyphenolic extract, hop bitter acid(MHBAs), gallotannin, Polygonum odoratum and Morus alba(MP), Yam extract, Bacopa monnieri extract(BME), Eriobotrya japonica extract(ELEJ), Phyto Meal extract(PM-EE), Pinus radiata bark extract(PRBE), *Centella asiatica* extract(CA), Wild Green Oat extract(WGOE), root of Polygala tenuifolia Willdenow(RPTW), Anredera cordifolia leaf (AC), Cistanche + Ginkgo biloba (CG).

**TABLE 2:** Characteristics of the studies included in the meta-analysis.

Author	Country	Year	Age	Total/male/female	BMI	Treatment	Control	Cognitive aspects
Ahles et al.	Netherlands	2020	I^1^:53.0(1.0) I^2^:53.0(1.0) C:53.0(1.0)	I^1^:34/11/23 I^2^: 35/11/24 C: 32/14/18	I^1^:29.5(0.4) I^2^:29.4(0.5) C: 29.3(0.5)	I^1^:Aronia melanocarpa Extract Duration:24 weeks Dose:90 mg/day I^2^:Aronia melanocarpa Extract Duration:24 weeks Dose:150 mg/day	Placebo	Cognitive flexibility Attention
Araki et al.	Japan	2020	NA	I:8/NA/NA C:13/NA/NA	NA	I: Rosemary extract Duration:4 weeks Dose:1g/day	Placebo	Global state Cognitive flexibility Executive function Memory Attention
Bell et al.	United Kingdom	2022	I:20.87(3.03) C:21.07(2.41)	I:30/6/24 C:30/3/27	I:22.72(3.72) C:21.42(3.66)	I: Grape seed polyphenol extract Duration:12 weeks Dose: 400 mg/day	Placebo	Executive function Memory
,Bensalem et al.	France	2019	NA	I:91/NA/NA C:98/NA/NA	NA	I: Polyphenols Duration:24 weeks Dose:600 mg/day	Placebo	Memory
Calabrese et al.	Portland	2008	NA	I:24/NA/NA C:24/NA/NA	NA	I: Bacopa monnieri Extract Duration:12 weeks Dose:300 mg/day	Placebo	Cognitive flexibility Memory
Chai et al.	United States	2019	I:70.0(3.7) C:69.5(3.9)	I:20/8/12 C:17/9/8	I:28.5(3.7) C:27.5(4.2)	I: tart cherry juice Duration:12 weeks Dose: 300 mL/day	Placebo	Memory Attention
Chen et al.	China	2024	I:58.8(11.3) C:61.1(7.39)	I:50/27/23 C: 50/27/23	I:23.7(3.08) C:23.3(2.62)	Cistanche extract + G. biloba extract Duration:12 weeks Dose:300 mg/day+120 mg/day	Placebo	Global state Cognitive flexibility Executive function Memory Attention
Cheng et al.	France	2024	I:71.02(2.03) C: 71.02(2.03)	I:45/18/27 C:45/18/27	I:25.05(2.95) C: 25.05(2.95)	Wild Blueberry Extract Duration:2 h Dose:222 mg	Placebo	Executive function Memory
Choi et al.	Korea	2016	I:18.25(0.84) C:18.2(0.76)	I:40/20/20 C: 40/21/19	I:21.17(2.80) C: 20.96(1.92)	Eriobotrya japonica Extract Duration:12 weeks Dose:750 mg/day	Placebo	Global state Memory
Cieza et al.	Germany	2003	I:55.9(3.8) C:56.8(3.4)	I:34/14/20 C:32/15/17	NA	G. biloba extract Duration:4 weeks Dose:240 mg/day	Placebo	Memory Attention
Crews et al.	United States	2005	I:69.17(7.11) C:69.39(5.80)	I:24/NA/NA C:23/NA/NA	NA	Cranberry Juice Duration:6 weeks Dose:32 ounces/day	Placebo	Executive function Memory Attention
Fukuda et al.	Japan	2020	I:54.6(5.4) C:55.4(5.3)	I:27/13/14 C:30/14/16	NA	Matured hop bitter acids Duration:12 weeks Dose:35 mg/day	Placebo	Cognitive flexibility Memory Attention
Fukuda et al.	Japan	2020	I:54.6(6.3) C:53.3(4.9)	I:49/20/29 C:49/21/28	NA	Matured hop bitter acids Duration:12 weeks Dose:35 mg/day	Placebo	Memory Attention
Hashimoto et al.	Japan	2022	I:67.0(1.2) C:68.9(1.4)	I:17/8/9 C:14/7/7	I:22.9(0.7) C:21.9(0.8)	Anredera cordifolia Duration:48 weeks Dose:1.12g/day	Placebo	Global state
Heuer et al.	United States	2023	I:33.65(8.84) C:33.15(10.15)	I:35/NA/NA C:33/NA/NA	I:27.51(4.07) C:27.47(5.19)	Ghala Rois extract Duration:6 weeks Dose:750 mg/day	Placebo	Executive function
Kaschel et al.	Germany	2011	NA	I:88/NA/NA C:89/NA/NA	NA	G. biloba extract Duration:6 weeks Dose:240 mg/day	Placebo	Memory
Lee et al.	Korea	2020	I:58.96(6.26) C:61.70(8.07)	I:26/3/23 C:27/5/22	NA	PhytoMeal-ethanol extract Duration:12 weeks Dose:600 mg/day	Placebo	Global state Executive function Memory
Mix et al.	United States	2002	I:66.97(6.12) C:68.60(6.96)	I:127/NA/NA C:122/NA/NA	NA	G. biloba extract Duration:6 weeks Dose:180 mg/day	Placebo	Memory
Noguchi-Shinohara et al.	Japan	2023	I:71.55(4.14) C:71.65(4.21)	I:162/56/106 C:161/57/104	NA	M. officinalis extract containing 500 mg of RA Duration:96 weeks Dose:500 mg/day	Placebo	Global state
Pipingas et al.	Australia	2008	I:58.2(4.2) C:58.4(4.0)	I:22/22/0 C:20/20/0	I:31.2(7.1) C:29.4(3.8)	Pinus radiata bark extract Duration:5weeks Dose:960 mg/day	Placebo	Memory Attention
Santos et al.	Brazil	2003	NA	I:23/23/0 C:25/25/0	NA	G. biloba extract Duration:32weeks Dose:80 mg/day	Placebo	Memory Attention
Shin et al.	Korea	2009	I:67.57(6.36) C:69.92(5.81)	I:28/6/22 C:25/2/23	NA	Roots of Polygala tenuifolia Willdenow Duration:8weeks Dose:300 mg/day	Placebo	Global state Executive function Memory Attention
Tohda et al.	Japan	2017	I:46.50(18.67) C:46.50(18.67)	I:28/12/16 C:28/12/16	NA	Yam Extract Duration:12weeks Dose:50 mg/day	Placebo	Global state
Wattanathorn et al.	Thailand	2008	I^1^:67.25(1.39) I^2^:62.00(4.34) I^3^:64.75(2.71) C:65.88(5.11)	I^1^:7/1/6 I^2^:7/1/6 I^3^:7/1/6 C:7/1/6	NA	I^1^:*Centella asiatica* Duration:8 weeks Dose:250 mg/day I^2^: *Centella asiatica* Duration:8 weeks Dose:500 mg/day I^3^: *Centella asiatica* Duration:8 weeks Dose:750 mg/day	Placebo	Memory Attention
Wattanathorn et al.	Thailand	2022	I^1^:50.47(3.20) I^2^:50.47(3.64) C:51.41(4.21)	I^1^:15/0/15 I^2^:15/0/15 C:15/0/15	I^1^:25.23(3.52) I^2^:24.91(3.81) C:24.27(2.91)	I^1^:extract of Polygonum odoratum and Morus alba Duration:8 weeks Dose:50 mg/day I^2^:extract of Polygonum odoratum and Morus alba Duration:8 weeks Dose:1500 mg/day	Placebo	Memory Attention
Whyte et al.	United Kingdom	2018	NA	I:85/NA/NA C:27/NA/NA	NA	Wild blueberry extract Duration:12 weeks Dose:500/1000 mg/day	Placebo	Memory
Wong et al.	Australia	2012	I:67.0(4.9) C:67.0(4.9)	I:37/25/12 C:37/25/12	I:26.4(3.6) C:26.4(3.6)	Wild green oat extract Duration:12 weeks Dose:500/1000 mg/day	Placebo	Cognitive flexibility Executive function Attention

### 3.3 Risk of bias

**FIGURE 2:**
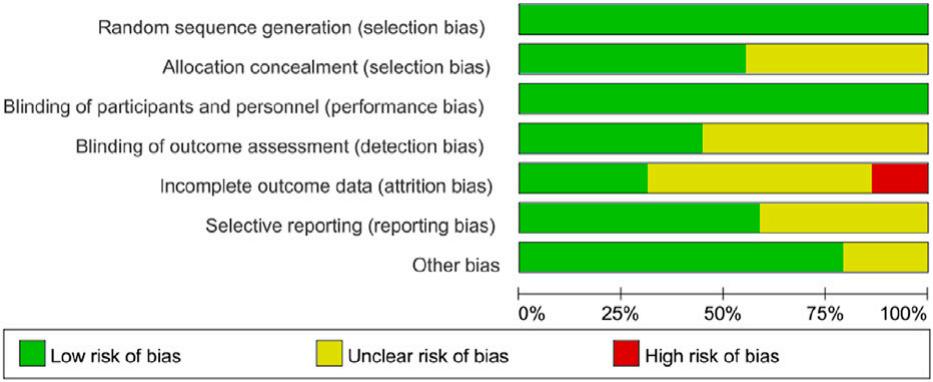
Risk of bias graph for RCTs.

All studies were considered to have a low risk of bias in generating random sequences and performance bias. 15 studies explicitly described allocation concealment and were therefore assessed as having a low risk of bias. 12 studies clearly defined the blinding of outcome assessors, indicating a low risk of bias. Regarding attrition bias, three studies showed a difference in the number of subjects before and after intervention (≥10 subjects), indicating a high risk of bias. Additionally, 12 studies may have an unclear risk due to the lack of reporting on pre-registered plans. Furthermore, six studies may have other risks of bias. The risk of bias assessment for the included studies is summarized in Figure 2.

### 3.4 Effects of natural extract on global cognitive state

**FIGURE 3:**
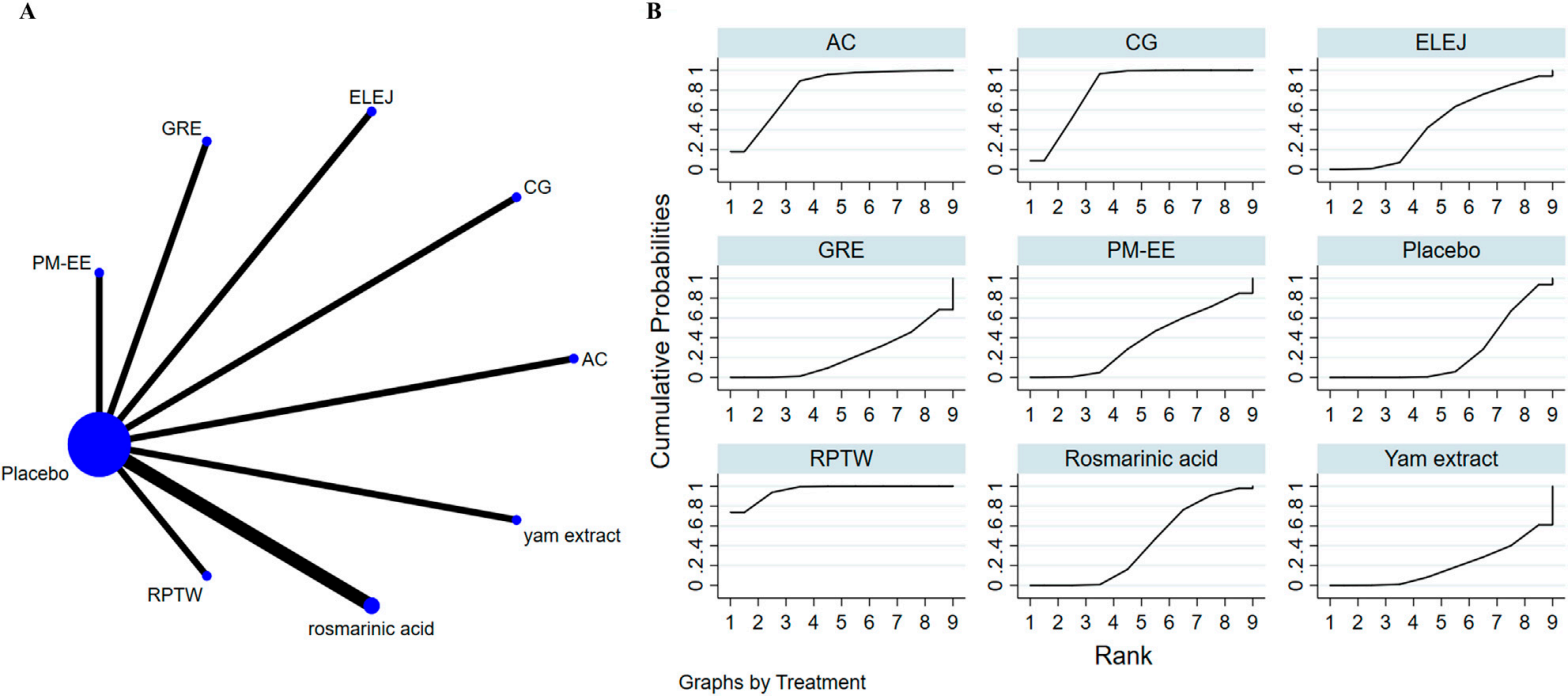
The evidence of natural extract to effect global cognitive state.

Nine studies assessed overall cognitive function. Results showed that RPTW, CG, and AC extracts were better than the placebo group in improving overall cognitive level, and the differences were statistically significant: RPTW (SMD = 1.28, 95% CI: 0.69–1.88), CG (SMD = 0.84, 95% CI: 0.42–1.27), AC (SMD = 0.86, 95% CI: 0.11–1.60). In the ranking of the probability of different natural products improving overall cognitive level(as depicted in Figure 3B), RPTW ranked first (SUCRA: 95.9%). Table 3 shows a comparison of different interventions.

**TABLE 3:** Ranking of each treatment based on SUCRA values, and the league table for relative effects of all treatments pairs on global cognitive state.

SUCRA:95.9% RPTW								
0.44 (−0.29,1.18)	SUCRA:82.0% CG							
0.43 (−0.53,1.38)	−0.01 (−0.87,0.84)	SUCRA:81.5% AC						
1.09 (0.33,1.84)	0.64 (0.01,1.27)	0.66 (−0.22,1.53)	SUCRA:46.1% ELEJ					
1.17 (0.54,1.80)	0.73 (0.25,1.21)	0.74 (−0.03,1.51)	0.08 (−0.43,0.59)	SUCRA:41.1% RA				
1.18 (0.38,1.99)	0.74 (0.05,1.43)	0.75 (−0.16,1.67)	0.10 (−0.61,0.81)	0.01 (−0.57,0.59)	SUCRA:37.1% PM-EE			
1.28 (0.69,1.88)	0.84 (0.42,1.27)	0.86 (0.11,1.60)	0.20 (−0.26,0.66)	0.12 (−0.10,0.33)	0.10 (−0.44,0.64)	SUCRA:24.4% Placebo		
1.34 (0.57,2.10)	0.90 (0.26,1.53)	0.91 (0.03,1.79)	0.25 (−0.41,0.92)	0.17 (−0.35,0.69)	0.15 (−0.56,0.87)	0.05 (−0.42,0.53)	SUCRA:22.2% GRE	
1.38 (0.59,2.18)	0.94 (0.27,1.62)	0.96 (0.05,1.86)	0.30 (−0.40,1.00)	0.21 (−0.35,0.78)	0.20 (−0.55,0.95)	0.10 (−0.43,0.62)	0.05 (−0.66,0.75)	SUCRA:19.7% YE

Abbreviations: AC, anredera cordifolia leaf; CG, Cistanche + Ginkgo biloba; ELEJ, eriobotrya japonica extract; GRE, ghala rois extract; PM-EE, phyto meal extract; RPTW, roots of Polygala tenuifolia Willdenow; RA, rosmarinic acid; YE, yam extract.

### 3.5 Effects of natural extract on attention

**FIGURE 4:**
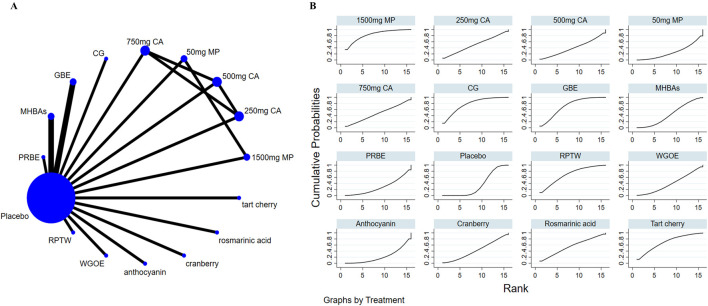
The evidence of natural extract to effect attention.

14 studies focused on attention. Results showed that no extract was observed to be superior to the placebo in improving attention. However, different doses of the extract MP demonstrated statistically significant differences in enhancing attention (SMD = −1.11, 95% CI: 2.09∼-0.12) (Table 4). In the ranking of the probability of different natural products improving attention, 1500 mg MP ranked first (SUCRA: 83.4%) (as depicted in Figure 4B).

**TABLE 4:** Ranking of each treatment based on SUCRA values, and the league table for relative effects of all treatments pairs on attention.

SUCRA:83.4% 1500 mg MP															
−0.20 (−1.43,1.04)	SUCRA:77.5% CG														
−0.33 (−1.47,0.80)	−0.14 (−1.10,0.82)	SUCRA:71.2% GBE													
−0.33 (−1.68,1.02)	−0.13 (−1.33,1.07)	0.00 (−1.10,1.11)	SUCRA:69.2% tart cherry												
−0.35 (−1.63,0.94)	−0.15 (−1.28,0.98)	−0.01 (−1.03,1.01)	−0.02 (−1.27,1.24)	SUCRA:69.0% RPTW											
−0.57 (−2.03,0.89)	−0.37 (−1.70,0.96)	−0.23 (−1.47,1.00)	−0.24 (−1.67,1.20)	−0.22 (−1.60,1.15)	SUCRA:55.6% RA										
−0.67 (−2.24,0.89)	−0.47 (−1.91,0.97)	−0.34 (−1.69,1.02)	−0.34 (−1.88,1.20)	−0.33 (−1.81,1.16)	−0.10 (−1.74,1.54)	SUCRA:50.2% 250 mg CA									
−0.74 (−2.30,0.83)	−0.54 (−1.98,0.90)	−0.40 (−1.76,0.96)	−0.41 (−1.95,1.13)	−0.39 (−1.87,1.09)	−0.17 (−1.81,1.47)	−0.07 (−1.29,1.16)	SUCRA:47.0% 750 mg CA								
−0.77 (−1.89,0.35)	−0.57 (−1.51,0.37)	−0.43 (−1.24,0.38)	−0.44 (−1.52,0.65)	−0.42 (−1.43,0.58)	−0.20 (−1.42,1.02)	−0.10 (−1.44,1.25)	−0.03 (−1.37,1.31)	SUCRA:44.4% MHBAs							
−0.80 (−2.10,0.49)	−0.61 (−1.75,0.54)	−0.47 (−1.50,0.57)	−0.47 (−1.74,0.79)	−0.46 (−1.65,0.74)	−0.24 (−1.62,1.15)	−0.13 (−1.63,1.36)	−0.07 (−1.56,1.42)	−0.04 (−1.05,0.98)	SUCRA:42.1% cranberry						
−0.86 (−2.42,0.70)	−0.66 (−2.10,0.78)	−0.52 (−1.88,0.83)	−0.53 (−2.07,1.01)	−0.51 (−2.00,0.97)	−0.29 (−1.93,1.35)	−0.19 (−1.41,1.04)	−0.12 (−1.35,1.10)	−0.09 (−1.44,1.25)	−0.06 (−1.55,1.43)	SUCRA:40.0% 500 mg CA					
−0.84 (−2.09,0.41)	−0.64 (−1.73,0.45)	−0.50 (−1.48,0.47)	−0.51 (−1.72,0.71)	−0.49 (−1.64,0.65)	−0.27 (−1.61,1.07)	−0.17 (−1.62,1.28)	−0.10 (−1.55,1.35)	−0.07 (−1.03,0.88)	−0.03 (−1.19,1.12)	0.02 (−1.43,1.47)	SUCRA:39.5% WGOE				
−0.89 (−1.86,0.09)	−0.69 (−1.45,0.07)	−0.55 (−1.14,0.04)	−0.56 (−1.49,0.38)	−0.54 (−1.38,0.30)	−0.32 (−1.41,0.77)	−0.22 (−1.44,1.01)	−0.15 (−1.37,1.07)	−0.12 (−0.68,0.44)	−0.08 (−0.94,0.77)	−0.03 (−1.25,1.20)	−0.05 (−0.83,0.73)	SUCRA:34.5% Placebo			
−1.05 (−2.36,0.26)	−0.85 (−2.01,0.31)	−0.71 (−1.77,0.34)	−0.72 (−2.00,0.56)	−0.70 (−1.92,0.51)	−0.48 (−1.88,0.92)	−0.38 (−1.88,1.13)	−0.31 (−1.82,1.19)	−0.28 (−1.32,0.75)	−0.25 (−1.47,0.98)	−0.19 (−1.69,1.31)	−0.21 (−1.38,0.96)	−0.16 (−1.04,0.71)	SUCRA:28.3% PRBE		
−1.11 (−2.09,-0.12)	−0.91 (−2.13,0.31)	−0.77 (−1.89,0.35)	−0.78 (−2.11,0.56)	−0.76 (−2.03,0.51)	−0.54 (−1.99,0.91)	−0.44 (−1.99,1.12)	−0.37 (−1.92,1.18)	−0.34 (−1.45,0.77)	−0.30 (−1.58,0.98)	−0.25 (−1.80,1.30)	−0.27 (−1.50,0.96)	−0.22 (−1.18,0.74)	−0.06 (−1.35,1.24)	SUCRA:25.9% 50 mg MP	
−1.15 (−2.41,0.11)	−0.95 (−2.05,0.15)	−0.81 (−1.80,0.18)	−0.82 (−2.04,0.41)	−0.80 (−1.96,0.35)	−0.58 (−1.93,0.77)	−0.48 (−1.94,0.98)	−0.41 (−1.87,1.05)	−0.38 (−1.35,0.59)	−0.34 (−1.51,0.82)	−0.29 (−1.75,1.17)	−0.31 (−1.42,0.80)	−0.26 (−1.06,0.54)	−0.10 (−1.28,1.08)	−0.04 (−1.28,1.20)	SUCRA:22.2% anthocyanin

Abbreviations: MP, polygonum odoratum and morus alba; CA, *centella asiatica*; CG, Cistanche + Ginkgo biloba; GBE, ginkgo biloba extract; MHBAs, matured hop extract; PRBE, pinus radiata bark extract; RPTW, roots of Polygala tenuifolia Willdenow; WGOE, wild green oat extract; RA, rosmarinic acid.

### 3.6 Effects of natural extract on memory

**FIGURE 5:**
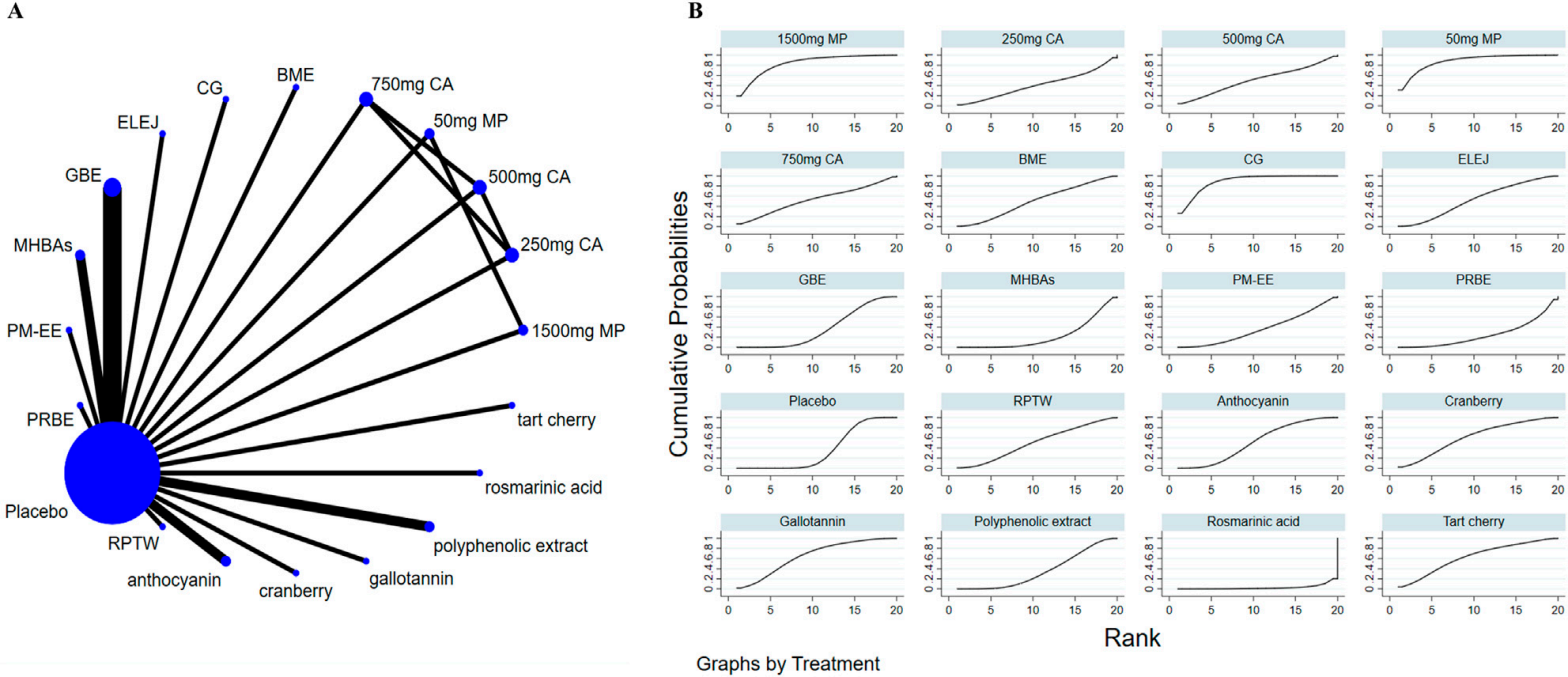
The evidence of natural extract to effect memory.

22 studies evaluated memory. Results showed that two natural extract interventions, CG and 50 mg MP, were better than placebo group in improving memory, and the differences were statistically significant: CG (SMD = 0.87, 95% CI: 0.29–1.45), 50 mg MP (SMD = 0.91, 95% CI: 0.07–1.76). In the ranking of the probability of different natural products improving memory, CG ranked first (SUCRA: 89.3%) (as depicted in Figure 5B). Table 5 shows a comparison of different interventions.

**TABLE 5:** Ranking of each treatment based on SUCRA values, and the league table for relative effects of all treatments pairs on memory.

SUCRA 89.3% CG																			
−0.05 (−1.07, 0.98)	SUCRA 88.1% 50 mg MP																		
0.06 (−0.96, 1.08)	0.11 (−0.71, 0.92)	SUCRA 84.2% 1500 mg MP																	
0.49 (−0.36, 1.34)	0.54 (−0.51, 1.58)	0.43 (−0.61, 1.47)	SUCRA 65.3% gallotannin																
0.49 (−0.48, 1.47)	0.54 (−0.61, 1.69)	0.43 (−0.71, 1.58)	0.00 (−1.00, 1.00)	SUCRA 63.1% tart cherry															
0.52 (−0.38, 1.43)	0.57 (−0.52, 1.66)	0.47 (−0.62, 1.55)	0.03 (−0.90, 0.97)	0.03 (−1.02, 1.08)	SUCRA 61.7% cranberry														
0.65 (−0.61, 1.91)	0.70 (−0.70, 2.10)	0.59 (−0.81, 1.99)	0.16 (−1.12, 1.44)	0.16 (−1.21, 1.52)	0.12 (−1.20, 1.44)	SUCRA 53.3% 750 mg CA													
0.67 (−0.17, 1.51)	0.72 (−0.32, 1.75)	0.61 (−0.42, 1.64)	0.18 (−0.69, 1.05)	0.18 (−0.82, 1.17)	0.15 (−0.78, 1.07)	0.02 (−1.25, 1.30)	SUCRA 53.2% ELEJ												
0.70 (−0.02, 1.41)	0.74 (−0.19, 1.68)	0.64 (−0.29, 1.57)	0.21 (−0.54, 0.95)	0.20 (−0.68, 1.09)	0.17 (−0.64, 0.98)	0.05 (−1.15, 1.24)	0.03 (−0.71, 0.76)	SUCRA 52.0% anthocyanin											
0.68 (−0.58, 1.95)	0.73 (−0.67, 2.13)	0.62 (−0.77, 2.02)	0.19 (−1.09, 1.47)	0.19 (−1.18, 1.56)	0.16 (−1.16, 1.48)	0.03 (−1.09, 1.15)	0.01 (−1.26, 1.29)	−0.01 (−1.21, 1.18)	SUCRA 51.1% 500 mg CA										
0.70 (−0.20, 1.60)	0.75 (−0.34, 1.83)	0.64 (−0.44, 1.72)	0.21 (−0.72, 1.14)	0.20 (−0.84, 1.25)	0.17 (−0.81, 1.15)	0.05 (−1.27, 1.36)	0.03 (−0.89, 0.95)	0.00 (−0.80, 0.80)	0.01 (−1.30, 1.33)	SUCRA 50.7% BME									
0.69 (−0.20, 1.57)	0.74 (−0.34, 1.81)	0.63 (−0.44, 1.70)	0.20 (−0.71, 1.11)	0.19 (−0.84, 1.22)	0.16 (−0.80, 1.13)	0.04 (−1.27, 1.34)	0.02 (−0.89, 0.92)	−0.01 (−0.79, 0.78)	0.00 (−1.30, 1.31)	−0.01 (−0.97, 0.95)	SUCRA 50.7% RPTW								
0.85 (−0.41, 2.11)	0.90 (−0.50, 2.30)	0.79 (−0.61, 2.19)	0.36 (−0.92, 1.64)	0.36 (−1.01, 1.72)	0.32 (−0.99, 1.64)	0.20 (−0.92, 1.32)	0.18 (−1.09, 1.45)	0.15 (−1.04, 1.35)	0.17 (−0.95, 1.29)	0.15 (−1.16, 1.47)	0.16 (−1.14, 1.46)	SUCRA 41.2% 250 mg CA							
0.89 (0.01, 1.78)	0.94 (−0.13, 2.01)	0.83 (−0.24, 1.90)	0.40 (−0.51, 1.31)	0.40 (−0.63, 1.43)	0.37 (−0.60, 1.33)	0.24 (−1.06, 1.55)	0.22 (−0.68, 1.12)	0.20 (−0.59, 0.98)	0.21 (−1.10, 1.51)	0.19 (−0.77, 1.15)	0.20 (−0.74, 1.15)	0.04 (−1.26, 1.34)	SUCRA 36.9% PM-EE						
0.87 (0.23, 1.51)	0.92 (0.03, 1.80)	0.81 (−0.07, 1.69)	0.38 (−0.30, 1.06)	0.37 (−0.46, 1.21)	0.34 (−0.41, 1.09)	0.22 (−0.93, 1.37)	0.20 (−0.47, 0.87)	0.17 (−0.32, 0.67)	0.19 (−0.97, 1.34)	0.17 (−0.57, 0.91)	0.18 (−0.54, 0.90)	0.02 (−1.13, 1.17)	−0.02 (−0.74, 0.70)	SUCRA 36.6% GBE					
0.88 (0.18, 1.58)	0.92 (−0.00, 1.85)	0.82 (−0.10, 1.74)	0.39 (−0.34, 1.12)	0.38 (−0.49, 1.26)	0.35 (−0.45, 1.15)	0.23 (−0.96, 1.41)	0.21 (−0.51, 0.93)	0.18 (−0.38, 0.75)	0.19 (−0.99, 1.38)	0.18 (−0.61, 0.97)	0.19 (−0.58, 0.96)	0.03 (−1.16, 1.21)	−0.01 (−0.79, 0.76)	0.01 (−0.47, 0.48)	SUCRA 36.1% PE				
0.87 (0.29, 1.45)	0.91 (0.07, 1.76)	0.81 (−0.03, 1.64)	0.38 (−0.24, 1.00)	0.37 (−0.41, 1.16)	0.34 (−0.35, 1.04)	0.22 (−0.90, 1.34)	0.20 (−0.41, 0.81)	0.17 (−0.24, 0.58)	0.18 (−0.94, 1.30)	0.17 (−0.52, 0.86)	0.18 (−0.49, 0.85)	0.02 (−1.10, 1.14)	−0.02 (−0.69, 0.64)	−0.00 (−0.28, 0.27)	−0.01 (−0.40, 0.38)	SUCRA 36.0% Placebo			
1.08 (0.16, 2.01)	1.13 (0.02, 2.24)	1.02 (−0.08, 2.13)	0.59 (−0.36, 1.55)	0.59 (−0.48, 1.66)	0.56 (−0.45, 1.56)	0.43 (−0.90, 1.77)	0.41 (−0.53, 1.36)	0.39 (−0.44, 1.22)	0.40 (−0.93, 1.73)	0.39 (−0.61, 1.39)	0.40 (−0.59, 1.38)	0.24 (−1.10, 1.57)	0.19 (−0.79, 1.18)	0.22 (−0.56, 0.99)	0.21 (−0.61, 1.03)	0.22 (−0.51, 0.94)	SUCRA 25.2% PRBE		
1.07 (0.35, 1.79)	1.11 (0.17, 2.05)	1.01 (0.07, 1.94)	0.58 (−0.18, 1.33)	0.57 (−0.32, 1.46)	0.54 (−0.28, 1.36)	0.42 (−0.78, 1.61)	0.40 (−0.35, 1.14)	0.37 (−0.22, 0.96)	0.38 (−0.82, 1.58)	0.37 (−0.44, 1.18)	0.38 (−0.41, 1.17)	0.22 (−0.98, 1.41)	0.17 (−0.62, 0.96)	0.20 (−0.31, 0.70)	0.19 (−0.39, 0.76)	0.20 (−0.23, 0.62)	−0.02 (−0.86, 0.82)	SUCRA 22.4% MHBAs	
1.94 (0.75, 3.12)	1.99 (0.66, 3.32)	1.88 (0.55, 3.21)	1.45 (0.24, 2.65)	1.45 (0.15, 2.74)	1.41 (0.17, 2.66)	1.29 (−0.23, 2.81)	1.27 (0.07, 2.47)	1.24 (0.13, 2.35)	1.26 (−0.27, 2.78)	1.24 (−0.00, 2.48)	1.25 (0.02, 2.48)	1.09 (−0.43, 2.61)	1.05 (−0.18, 2.28)	1.07 (0.00, 2.14)	1.06 (−0.04, 2.16)	1.07 (0.04, 2.10)	0.86 (−0.41, 2.12)	0.87 (−0.24, 1.99)	SUCRA 2.9% RA

Abbreviations: MP, polygonum odoratum and morus alba; CA, *centella asiatica*; BME, bacopa monnieri extract; CG, Cistanche + Ginkgo biloba; ELEJ, eriobotrya japonica extract; GBE, ginkgo biloba extract; MHBAs, matured hop extract; PM-EE, phyto meal extract; PRBE, pinus radiata bark extract; RPTW, roots of Polygala tenuifolia Willdenow; PE, polyphenolic extract; RA, rosmarinic acid.

### 3.7 Effects of natural extract on executive function

**FIGURE 6:**
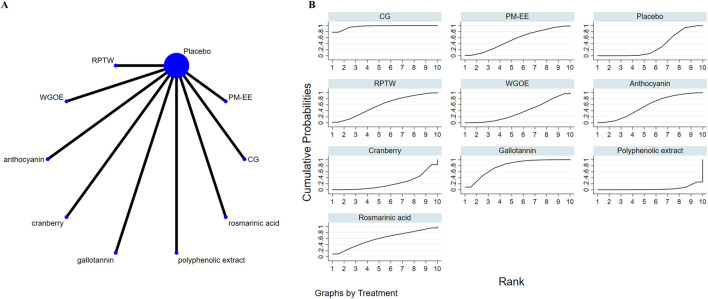
The evidence of natural extract to effect executive function.

Nine studies investigated executive function. Results showed that two natural extract, CG and gallotannin, were better than placebo group in improving executive function, and the differences were statistically significant: CG (SMD = −0.93, 95% CI: 1.36∼-0.50), gallotannin (SMD = −0.53, 95% CI: 1.01∼-0.04). In the ranking of the probability of different natural products improving executive function, CG ranked first (SUCRA: 96.9%) (as depicted in Figure 6B). Table 6 shows a comparison of different interventions.

**TABLE 6:** Ranking of each treatment based on SUCRA values, and the league table for relative effects of all treatments pairs on executive function.

SUCRA:96.9% CG									
−0.40 (−1.05,0.25)	SUCRA:78.2% gallotannin								
−0.61 (−1.60,0.38)	−0.21 (−1.22,0.80)	SUCRA:60.1% RA							
−0.67 (−1.36,0.02)	−0.27 (−1.00,0.46)	−0.06 (−1.10,0.98)	SUCRA:57.9% RPTW						
−0.71 (−1.30,-0.11)	−0.31 (−0.94,0.33)	−0.10 (−1.07,0.88)	−0.04 (−0.72,0.65)	SUCRA:55.9% anthocyanin					
−0.71 (−1.40,-0.02)	−0.31 (−1.04,0.41)	−0.10 (−1.14,0.94)	−0.04 (−0.81,0.72)	−0.01 (−0.69,0.68)	SUCRA:54.4% PM-EE				
−0.91 (−1.54,-0.29)	−0.51 (−1.18,0.15)	−0.30 (−1.30,0.69)	−0.24 (−0.95,0.46)	−0.21 (−0.82,0.41)	−0.20 (−0.91,0.51)	SUCRA:37.0% WGOE			
−0.93 (−1.36,-0.50)	−0.53 (−1.01,-0.04)	−0.32 (−1.20,0.57)	−0.26 (−0.80,0.28)	−0.22 (−0.64,0.19)	−0.22 (−0.76,0.32)	−0.01 (−0.47,0.44)	SUCRA:33.2% Placebo		
−1.12 (−1.83,-0.40)	−0.72 (−1.47,0.03)	−0.51 (−1.56,0.55)	−0.45 (−1.24,0.34)	−0.41 (−1.12,0.30)	−0.41 (−1.19,0.38)	−0.20 (−0.94,0.53)	−0.19 (−0.76,0.38)	SUCRA:22.1% cranberry	
−1.46 (−2.13,-0.78)	−1.06 (−1.76,-0.35)	−0.85 (−1.87,0.18)	−0.79 (−1.53,-0.04)	−0.75 (−1.41,-0.09)	−0.74 (−1.49,0.00)	−0.54 (−1.23,0.15)	−0.53 (−1.04,-0.01)	−0.34 (−1.11,0.43)	SUCRA:4.3% PE

Abbreviations: CG, Cistanche + Ginkgo biloba; PM-EE, phyto meal extract; RPTW, roots of Polygala tenuifolia Willdenow; WGOE, wild green oat extract; PE, polyphenolic extract; RA, rosmarinic acid.

### 3.8 Effects of natural extract on cognitive flexibility

**FIGURE 7:**
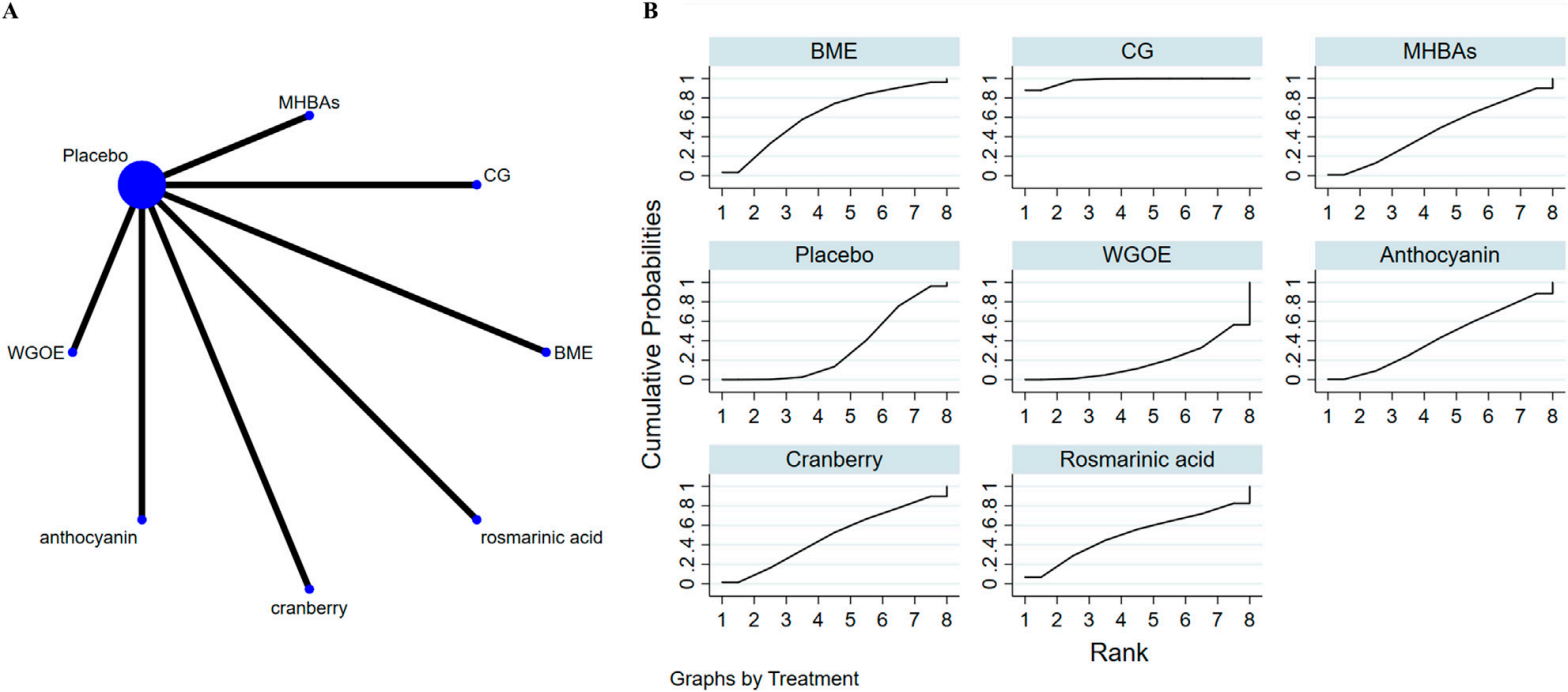
The evidence of natural extract to effect cognitive flexibility.

Seven studies assessed cognitive flexibility. Results showed that CG was better than placebo group in improving cognitive flexibility, and the differences was statistically significant (SMD = −0.94, 95% CI: 1.37∼-0.50). In the ranking of the probability of different natural products improving cognitive flexibility, CG ranked first (SUCRA: 98.0%) (as depicted in Figure 7B). Table 7 shows a comparison of different interventions.

**TABLE 7:** Ranking of each treatment based on SUCRA values, and the league table for relative effects of all treatments pairs on cognitive flexibility.

SUCRA:98.0% CG							
−0.64 (−1.36,0.07)	SUCRA:62.7% BME						
−0.76 (−1.74,0.22)	−0.12(-1.17,0.93)	SUCRA:50.6% RA					
−0.80(-1.52,-0.08)	−0.15(-0.96,0.65)	−0.04(-1.09,1.02)	SUCRA:48.4% cranberry				
−0.82(-1.50,-0.14)	−0.17(-0.95,0.60)	−0.06(-1.08,0.97)	−0.02 (−0.79,0.75)	SUCRA:46.5% MHBAs			
−0.87(-1.51,-0.22)	−0.22(-0.97,0.53)	−0.10(-1.11,0.90)	−0.07 (−0.82,0.68)	−0.05 (−0.76,0.66)	SUCRA:42.7% anthocyanin		
−0.94(-1.37,-0.50)	−0.29(-0.86,0.28)	−0.17(-1.06,0.71)	−0.14 (−0.71,0.44)	−0.12 (−0.64,0.40)	−0.07 (−0.55,0.41)	SUCRA:32.7% Placebo	
−1.10(-1.73,-0.47)	−0.45(-1.18,0.28)	−0.34(-1.33,0.66)	−0.30 (−1.03,0.43)	−0.28 (−0.97,0.41)	−0.23 (−0.90,0.43)	−0.16 (−0.62,0.29)	SUCRA:18.2% WGOE

Abbreviations: BME, bacopa monnieri extract; CG, Cistanche + Ginkgo biloba; MHBAs, matured hop extract; WGOE, wild green oat extract; RA, rosmarinic acid.

### 3.9 Funnel plot characteristics

**FIGURE 8:**
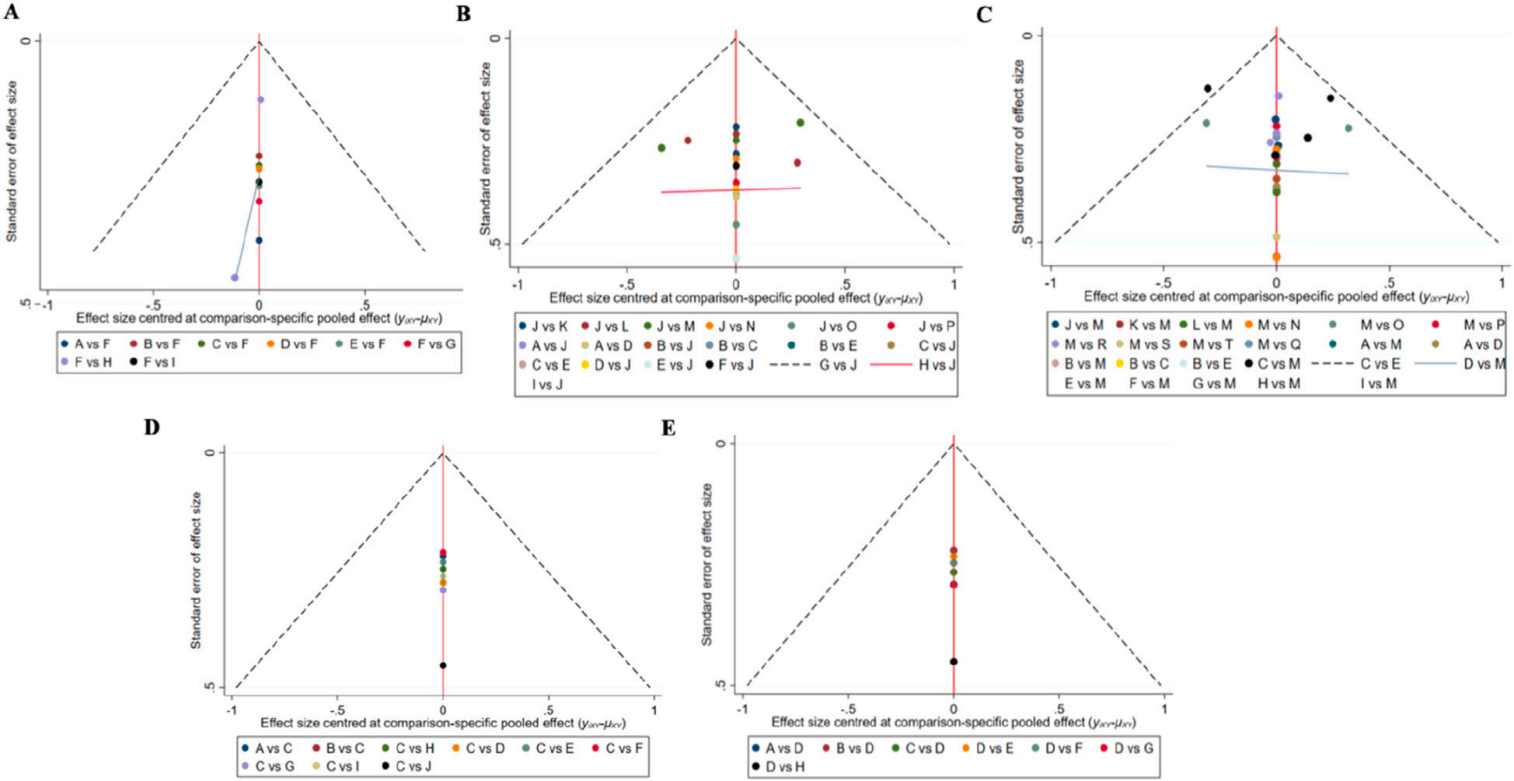
Funnel plot on publication bias. **(A)**: global cognitive state; **(B)**: attention; **(C)**: memory; **(D)**: executive function; **(E)**: cognitive flexibility.

Independent funnel plots were constructed for each outcome measure to investigate the possibility of publication bias. Visual inspection of the funnel plots did not reveal any obvious publication bias. For detailed information, see Figure 8.The p-values for the consistency and inconsistency tests of direct and indirect comparisons between the studies are all greater than 0.05, indicating consistency among the studies. For comprehensive details, please refer to Supplementary Table S1.

## 4 Discussion

In this study, we compared the efficacy of various natural extracts in enhancing cognitive function. A total of 27 randomized controlled trials were evaluated, involving 19 distinct natural extracts and a substantial sample of 2,334 participants. The results indicated that the roots of Polygala tenuifolia Willdenow, Cistanche tubulosa combined with Ginkgo biloba, and the leaves of Anredera cordifolia significantly improved cognitive function in healthy adults. Among these, Cistanche tubulosa combined with Ginkgo biloba was the most effective in enhancing memory, executive function, and cognitive flexibility. Additionally, compared to the placebo, MP improved attention in healthy individuals. In conclusion, our findings suggest that Cistanche tubulosa combined with Ginkgo biloba may be the most suitable natural extract for enhancing cognitive function in healthy adults.

In our study, nine pieces of literature evaluated the overall cognitive levels of the samples. The survey methods employed several widely used assessment tools, including the MMSE, MoCA, ADAS, and Cognitrax tests. Among all interventions, the three most effective were Polygala tenuifolia Willdenow, Cistanche tubulosa combined with Ginkgo biloba, and Anredera cordifolia leaves. The roots of Polygala tenuifolia Willdenow have been traditionally used for their beneficial effects on insomnia, anxiety, restlessness, and memory in humans(Jin et al., 2014; Park et al., 2019; Lee et al., 2009). RPTW can influence cognition through both direct and indirect effects. Specifically, RPTW enhances cognitive function through several biological pathways. Firstly, it improves glucose metabolism in the brain, which is crucial for maintaining cognitive function, especially since cognitive decline is often associated with impaired glucose utilization(Park et al., 2002). Secondly, RPTW extracts inhibit acetylcholinesterase activity, thereby increasing acetylcholine levels, which plays a significant role in improving memory and learning(Shin et al., 2009b). Additionally, active compounds in RPTW, such as tenuifolin, Yuanzhi-1, tenuigenin, and tenuifoliside, possess anti-depressant, anti-inflammatory, and anti-seizure properties, contributing to neuroprotection and reducing neuroinflammation, which helps mitigate cognitive decline(Jin et al., 2014; Shin et al., 2009b). Lastly, RPTW also protects the nervous system by reducing oxidative stress and inflammation, which are critical factors in neurodegeneration(Cao et al., 2016; Shi et al., 2015). These active substances indirectly affect cognitive function through a variety of protective mechanisms.

The study results also indicate that Cistanche tubulosa combined with Ginkgo biloba can significantly improve overall cognitive levels, consistent with previous research. A meta-analysis of 21 trials involving 2,608 patients with mild cognitive impairment (MCI) or Alzheimer’s disease (AD) indicated that Ginkgo biloba combined with conventional medication was superior to conventional medication alone in improving MMSE scores at 24 weeks(Yang et al., 2016) Ginkgo biloba contains flavonoids and terpene lactones, which possess antioxidant properties. Animal experiments have demonstrated that Ginkgo biloba stabilizes the redox state of cells by upregulating reactive oxygen species (ROS)-related active enzymes(Shi et al., 2010b), increasing the activities of superoxide dismutase (SOD) and catalase (CAT) in the hippocampus of rats(Shi et al., 2010a), enhancing the activities of total superoxide dismutase (T-SOD), catalase (CAT), and glutathione peroxidase (GSH-Px) in neurons(Chen et al., 2019), and promoting the activity of glutathione reductase and γ-glutamylcysteine synthetase(Shi et al., 2010c). A cohort study involving patients with Alzheimer’s disease (AD) using herbal therapies containing Cistanche tubulosa demonstrated improvements in both short-term and long-term MMSE scores(Shi et al., 2017). *In vitro* studies have indicated that extracts from Cistanche tubulosa can protect dopaminergic neurons from hydrogen peroxide (H_2_O_2_)-induced oxidative damage and significantly increase the levels of nerve growth factor and brain-derived neurotrophic factor(Lin et al., 2013). Currently, no studies have compared the effects of the combined use of these two extracts with their individual uses, highlighting the need for larger-scale randomized controlled trial evidence to support this.

Anredera cordifolia has been used as a medicinal plant in East Asia for several centuries(Matsuzaki and Ohizumi, 2021; Hashimoto et al., 2020). In preclinical studies, AC has been shown to improve memory impairment in mice induced by the N-methyl-D-aspartate (NMDA) receptor antagonist MK-801. Interestingly, in a randomized controlled trial, Hashimoto et al. observed that AC improves cognitive function by reducing serum triglyceride and glucose levels(Hashimoto et al., 2022).

Cognitive performance is a broad concept that encompasses multiple complex processes, which can be categorized into six main areas: attention, executive function, perceptual-motor function, learning and memory, language, and social cognition(Sachdev et al., 2014). We focus on the first four areas by assessing subfields such as attention, executive function, cognitive flexibility, and delayed recall, which are commonly evaluated in comparable studies(Ashendorf et al., 2009; Van der Elst et al., 2006; Ahles et al., 2020; Fukuda et al., 2020a).

In terms of attention assessment, studies often employ the Digit Vigilance Task or the Trail Making Test (Part A), 1500  mg MP ranked first in different natural products improving attention. MP (Polygonum odoratum and Morus alba) are two culinary herbs widely consumed in Thai cuisine, with quercetin as the primary functional component. As a commonly used bioflavonoid, quercetin exhibits various pharmacological properties, including anti-inflammatory(Li et al., 2016), antioxidant(Costa et al., 2016), and anti-amyloidogenic effects(Barreca et al., 2016). Rishitha and Muthuraman (2018) reported the neuroprotective effects of quercetin in attention deficit disorders, alleviating oxidative stress and cell apoptosis in mouse brain tissue via the Keap1/Nrf2/HO-1 pathway, thereby improving attention(Cheng M. et al., 2024). Although no significant effects were observed for different doses of MP in improving attention, the results comparing 1,500 mg MP versus 50 mg MP (SMD = −1.11, 95% CI: 2.09-0.12) suggest that the dosage of MP may have a positive correlation with attention improvement.

In all subfields, we observed a positive effect of Cistanche tubulosa combined with Ginkgo biloba on the outcomes, consistent with the results for overall cognitive function. As previously mentioned regarding their individual mechanisms for improving cognitive function, studies indicate that combined interventions using the two extracts can provide benefits through synergistic effects, thereby increasing efficiency, reducing adverse reactions, enhancing stability or bioavailability, and lowering therapeutic doses(Sungkamanee et al., 2014; Wattanathorn et al., 2018). In this study, we observed that the combined intervention of CG was stronger in the subfields of memory and attention than the effects of Ginkgo biloba used alone (SMD = 0.87, 95% CI: 0.23–1.51; SMD = −0.14, 95% CI: 1.10–0.82), suggesting that the combined use of plant extracts may help improve cognitive function in healthy adults.

Our research results suggest that the application of natural products to improve cognitive function in healthy adults may lead to changes in lifestyle or a reduction in medication use. These findings also indicate that the use of natural products could potentially be incorporated into the management of cognitive decline, mild cognitive impairment, dementia, and Alzheimer’s disease in the future. Natural products demonstrate significant potential in the prevention, delay of progression, and treatment of cognitive decline. However, the beneficial use of natural products requires comprehensive consideration of various factors, including the assessment of cognitive status, selection of appropriate natural products, development of personalized treatment plans, monitoring of efficacy and side effects, lifestyle adjustments, and provision of psychological support. It is advisable to consult a physician or pharmacist before using natural products to ensure safety and effectiveness. The results of this study are preliminary and indicate the need for further research in this field, particularly in the form of high-quality randomized controlled trials with large sample sizes, rigorous designs, and long follow-up periods.

Our research is influenced by certain limitations. Although we attempted to control for the heterogeneity of the included studies, some level of heterogeneity between studies is unavoidable (e.g., differences in survey methods). Additionally, due to the limited relevant data provided in the studies, we were unable to analyze the adverse effects of the tested natural products. While natural products are generally known for their relatively mild side effects, this remains an area requiring further investigation. To address these limitations, we recommend that future studies improve methodological rigor by clearly reporting randomization and allocation concealment procedures, ensuring adequate blinding, and minimizing attrition with detailed explanations for participant dropouts. Furthermore, future research should explore dose-response relationships, conduct longer-term trials (e.g., 6–12 months), and adopt standardized cognitive assessment tools (e.g., MMSE, MoCA) to reduce heterogeneity and improve comparability across studies. Systematic monitoring of adverse effects and subgroup analyses based on age, baseline cognitive performance, and genetic factors should also be prioritized to identify populations that may benefit most from these interventions. In summary, considering the relatively small number of studies included in our research and the limited direct comparison evidence for some interventions, caution should be exercised when interpreting the results. However, by addressing these limitations, future studies can provide more robust and reliable evidence on the efficacy and safety of natural extracts for cognitive enhancement.

## 5 Conclusion

Our network meta-analysis identifies CG (Cistanche + Ginkgo biloba) as the most effective intervention for enhancing memory, executive function, and cognitive flexibility in healthy adults, with an optimal dosage of 300 mg/day Cistanche and 120 mg/day Ginkgo biloba. To further validate these findings and promote their clinical application, future studies should prioritize: (1) dose-response evaluations and long-term efficacy trials (6–12 months); (2) subgroup analyses based on age, baseline cognitive status, and genetic factors; (3) exploration of synergistic combinations with other natural extracts; and (4) standardized cognitive assessments (e.g., MMSE, MoCA) to ensure consistency across studies. These steps will strengthen the evidence base and facilitate the development of evidence-based recommendations for cognitive health.

## Data Availability

The original contributions presented in the study are included in the article/Supplementary Material, further inquiries can be directed to the corresponding author.
