# Supplementary material for: Effects of natural extracts in cognitive function of healthy adults: a systematic review and network meta-analysis
Source: Front Pharmacol. 2025 Mar 27;16:1573034. doi: 10.3389/fphar.2025.1573034 (PMC11982825; doi:10.3389/fphar.2025.1573034)
Supplement: Supplementary file 1 [file DataSheet1.pdf]

Side	Direct		Indirect		Difference			tau
	Coef.	Std. Err.	Coef.	Std. Err.	Coef.	Std. Err.	P>|z|	
AC VS Placebo	-.8565371	.3790914	-0.13645	110.0728	-0.72009	110.0735	0.995	1.73e-07
CG VS Placebo	-.8430746	.2181099	-0.87133	630.7036	0.028255	630.7037	1	4.35e-08
ELEJ VS Placebo	-.1989712	.2364166	-1.52922	628.4862	1.330248	628.4863	0.998	4.84e-06
GRE VS Placebo	.0521056	.2426848	-1.76437	631.4842	1.816475	631.4844	0.998	2.90e-07
PM-EE VS Placebo	-.1019523	.274966	-1.61169	628.2836	1.509737	628.2838	0.998	1.10e-07
RPTW VS Placebo	1.284597	.3046526	3.001335	666.7068	-1.71674	666.7067	0.998	1.30e-07
RA VS Placebo	.115026	.1081165	1.821899	473.9433	-1.70687	473.9432	0.997	2.11e-05
YE VS Placebo	-.0988487	.2674395	1.616306	640.0132	-1.715155	640.0132	0.998	2.74e-08

Supplementary Table 1-1. Consistency test for global cognitive state.

Abbreviations: AC, Anredera cordifolia leaf; CG, Cistanche + Ginkgo biloba; ELEJ, Eriobotrya japonica extract; GRE, Ghala Rois extract; PM-EE, Phyto Meal extract; RPTW, roots of Polygala tenuifolia Willdenow; RA, rosmarinic acid; YE, yam extract

Side	Direct		Indirect		Difference			tau
	Coef.	Std. Err.	Coef.	Std. Err.	Coef.	Std. Err.	P>|z|	
1500mg MP VS 50mg MP	1.107411	.5022878	-.3459371	55.51553	1.453348	55.51797	0.979	.3220669
1500mg MP VS Placebo	.8871471	.496826	.1604729	27.71182	.7266742	27.71627	0.979	.3220669
50mg MP VS Placebo	-.220264	.487507	-1.673612	55.48294	1.453348	55.48498	0.979	.322067
250mg CA VS Placebo	.2163581	.6249203	1.55771	199.8809	-1.341352	199.8833	0.995	.3220406
500mg CA VS Placebo	.0274688	.6240531	1.36848	199.8541	-1.341012	199.855	0.995	.3220407
750mg CA VS Placebo	.1500338	.6244629	1.491107	200.2134	-1.341073	200.2144	0.995	.3220408
CG VS Placebo	.6883995	.3872011	1.085455	199.9356	-.3970554	199.9362	0.998	.3220408
GBE VS Placebo	.5521497	.2996931	1.221713	141.5449	-.6695633	141.5455	0.996	.3220426
MHBAs VS Placebo	.1197987	.2832788	1.654111	141.5403	-1.534313	141.5408	0.991	.3220444
PRBE VS Placebo	-.1624783	.4466756	1.936364	200.3646	-2.098842	200.3655	0.992	.3220408
RPTW VS Placebo	-.5415138	.4271568	-2.3154	199.859	1.773886	199.8591	0.993	.3220407
WGOE VS Placebo	-.0482305	.3972167	-1.822102	200.4464	1.773872	200.4465	0.993	.3220408
anthocyanin VS Placebo	.2609906	.4061133	-1.512881	200.9838	1.773872	200.9839	0.993	.3220408
cranberry VS Placebo	-.0832154	.4346691	-1.855958	199.5413	1.772743	199.5413	0.993	.322041
rosmarinic acid VS Placebo	-.3184647	.5556542	-2.092344	200.3197	1.773879	200.3194	0.993	.3220408
tart cherry VS Placebo	-.5567933	.4760835	-2.330679	200.2152	1.773886	200.2151	0.993	.3220409

Supplementary Table 1-2. Consistency test for attention.

Abbreviations: MP, Polygonum odoratum and Morus alba; CA, Centella asiatica; CG, Cistanche + Ginkgo biloba; GBE, Ginkgo biloba extract; MHBAs, matured hop extract; PRBE, Pinus radiata bark extract; RPTW, roots of Polygala tenuifolia Willdenow; WGOE, wild green oat extract

Side	Direct		Indirect		Difference			tau
	Coef.	Std. Err.	Coef.	Std. Err.	Coef.	Std. Err.	P>|z|	
1500mg MP VS 50mg MP	.1064767	.4167672	1.66484	43.61742	-1.558363	43.61936	0.972	.200551
1500mg MP VS Placebo	-.8085371	.4262324	-.0293561	21.79059	-.779181	21.79476	0.971	.2005511
50mg MP VS Placebo	-.9150138	.4288977	.6432307	43.6646	-1.558245	43.66688	0.972	.2005504
250mg CA VS Placebo	-.0187572	.5709128	-1.597754	199.7862	1.578997	199.7885	0.994	.2005465
500mg CA VS Placebo	-.184442	.5716058	-1.763438	199.8962	1.578996	199.897	0.994	.2005465
750mg CA VS Placebo	-.2184017	.5718872	-1.797398	199.8655	1.578996	199.8663	0.994	.2005465
BME VS Placebo	-.1698211	.3519738	-1.44668	199.8786	1.276859	199.8793	0.995	.2005465
CG VS Placebo	-.8679309	.2967062	-.7487011	200.0905	-.1192298	200.091	1.000	.2005466
ELEJ VS Placebo	-.19797	.3100136	-1.418668	200.3164	1.220698	200.3169	0.995	.2005457
GBE VS Placebo	.000641	.139994	-1.617223	100.4522	1.617864	100.4524	0.987	.2005476
MHBAs VS Placebo	.1977008	.2165746	-1.81425	141.238	2.01195	141.2384	0.989	.2005467
PM-EE VS Placebo	.0236624	.3401811	-1.640217	199.9953	1.66388	199.996	0.993	.2005462
PRBE VS Placebo	.2163851	.3691908	-1.832901	199.9085	2.049286	199.9094	0.992	.2005465
RPTW VS Placebo	.1798264	.3409813	1.796352	199.7197	-1.616526	199.7196	0.994	.2005465
anthocyanin VS Placebo	.1721162	.2090764	1.788668	141.4827	-1.616551	141.4827	0.991	.2005469
cranberry VS Placebo	.3430291	.3560283	1.95954	200.6189	-1.616511	200.6188	0.994	.2005465
gallotannin VS Placebo	.3774371	.3165745	1.994011	198.8684	-1.616574	198.8683	0.994	.2005465
polyphenolic extract VS Placebo	-.0094437	.1975093	1.607083	141.0237	-1.616527	141.0237	0.991	.2005471
rosmarinic acid VS Placebo	-1.071441	.5267229	.5450689	200.1736	-1.616509	200.1731	0.994	.2005465
tart cherry VS Placebo	.3740814	.4003219	1.990601	200.5169	-1.616519	200.5167	0.994	.200547

Supplementary Table 1-3. Consistency test for memory.

Abbreviations: MP, Polygonum odoratum and Morus alba; CA, Centella asiatica; BME, Bacopa monnieri extract; CG, Cistanche + Ginkgo biloba; ELEJ, Eriobotrya japonica extract; GBE, Ginkgo biloba extract; MHBAs, matured hop extract; PM-EE, Phyto Meal extract; PRBE, Pinus radiata bark extract; RPTW, roots of Polygala tenuifolia Willdenow

Side	Direct		Indirect		Difference			tau
	Coef.	Std. Err.	Coef.	Std. Err.	Coef.	Std. Err.	P>|z|	
CG VS Placebo	.92705	.2199271	.0270035	35.31514	.9000465	35.31582	0.980	.0000126
PM-EE VS Placebo	.2160334	.2756816	1.636951	.2756816	-1.420917	0		2.52e-06
RPTW VS Placebo	-.2579069	.2838034	-2.110789	199.732	1.852882	199.7318	0.993	.0635352
WGOE VS Placebo	-.0144496	.2351441	-1.867296	201.5311	1.852846	201.5311	0.993	.0351373
anthocyanin VS Placebo	-.2219394	.2178397	-2.07483	199.8762	1.852891	199.8761	0.993	.0507648
cranberry VS Placebo	.1901415	.2933769	-1.662782	606.2562	1.852924	606.2428	0.998	.0172608
gallotannin VS Placebo	-.5273758	.2515134	-2.380233	201.4452	1.852858	201.445	0.993	.0467601
polyphenolic extract VS Placebo	.5286864	.2630896	-1.562382	347.4	2.091068	347.3962	0.995	.0002191
rosmarinic acid VS Placebo	-.3171945	.4549352	-2.170026	200.0126	1.852831	200.0121	0.993	.0441778

Supplementary Table 1-4. Consistency test for executive function.

Abbreviations: CG, Cistanche + Ginkgo biloba; PM-EE, Phyto Meal extract; RPTW, roots of Polygala tenuifolia Willdenow; WGOE, wild green oat extract

Side	Direct		Indirect		Difference			tau
	Coef.	Std. Err.	Coef.	Std. Err.	Coef.	Std. Err.	P>|z|	
BME VS Placebo	.2913688	.2903317	.1754502	40.78732	.1159186	40.78836	0.998	3.93e-06
CG VS Placebo	.9358937	.2202844	-.3534013	.2202844	1.289295	0		2.13e-06
MHBAs VS Placebo	.1168393	.2655356	.4658086	.2655356	-.3489693	0		3.69e-06
WGOE VS Placebo	.1621806	.2329479	-.4204623	203.164	.5826429	203.1644	0.998	.0025741
anthocyanin VS Placebo	-.0705831	.2470393	-.6532328	1027.396	.5826496	1027.378	1.000	.0005658
cranberry VS Placebo	-.1372415	.2921916	-.719901	199.8693	.5826596	199.8692	0.998	.0010466
rosmarinic acid VS Placebo	-.1740536	.4504003	-.7567035	205.2593	.58265	205.2583	0.998	.0005731

Supplementary Table 1-5. Consistency test for cognitive flexibility.

Abbreviations: BME, Bacopa monnieri extract; CG, Cistanche + Ginkgo biloba; MHBAs, matured hop extract; WGOE, wild green oat extract
